# Influence of Individual and Contextual Perceptions and of Multiple Neighborhoods on Depression

**DOI:** 10.3390/ijerph17061958

**Published:** 2020-03-17

**Authors:** Médicoulé Traoré, Cécile Vuillermoz, Pierre Chauvin, Séverine Deguen

**Affiliations:** 1 INSERM, Sorbonne Université, Institut Pierre Louis d’Épidémiologie et de Santé Publique, IPLESP, Department of social epidemiology, F75012 Paris, France; cecile.vuillermoz@inserm.fr (C.V.); pierre.chauvin@inserm.fr (P.C.); severine.deguen@ehesp.fr (S.D.); 2 EHESP School of Public Health, F35043 Rennes, France

**Keywords:** depression, cumulative exposure score, contextual perceptions, multilevel analysis, neighborhood, daily mobility, life course, social inequalities

## Abstract

The risk of depression is related to multiple various determinants. The consideration of multiple neighborhoods daily frequented by individuals has led to increased interest in analyzing socio-territorial inequalities in health. In this context, the main objective of this study was (i) to describe and analyze the spatial distribution of depression and (ii) to investigate the role of the perception of the different frequented spaces in the risk of depression in the overall population and in the population stratified by gender. Data were extracted from the 2010 SIRS (a French acronym for “health, inequalities and social ruptures”) cohort survey. In addition to the classic individual characteristics, the participants reported their residential neighborhoods, their workplace neighborhoods and a third one: a daily frequented neighborhood. A new approach was developed to simultaneously consider the three reported neighborhoods to better quantify the level of neighborhood socioeconomic deprivation. Multiple simple and cross-classified multilevel logistic regression models were used to analyze the data. Depression was reported more frequently in low-income (OR = 1.89; CI = [1.07–3.35]) or middle-income (OR = 1.91; CI = [1.09–3.36]) neighborhoods and those with cumulative poverty (OR = 1.64; CI = [1.10–2.45]). In conclusion, a cumulative exposure score, such as the one presented here, may be an appropriate innovative approach to analyzing their effects in the investigation of socio-territorial inequalities in health.

## 1. Introduction

Depression continues to increase exponentially worldwide. The World Health Organization (WHO) estimates that mental disorders caused by depression are the primary risk factors leading to death or disability [1]. According to the WHO World Mental Health (WMH) Survey Initiative [2], France ranks first in the lifetime prevalence of major depressive episodes (21.0%) among the 18 countries that participate in the WMH surveys, ahead of the USA (19.2%), Brazil (Sao Paulo, 18.4%), the Netherlands (17.9%) and New Zealand (17.8%) [3]. In France, where the consumption of psychotropic drugs is four times higher than in other European countries [4], the prevalence of depression in the past 12 months in the 18–75-year-old population was 9.8% in 2017, and twice as high in women (13.0%) than in men (6.4%) [5]. In addition, the economic costs associated with depression are staggering: in 2007, the costs of depression in the European Economic Area amounted to €136.3 billion. The largest share of these costs stems from reduced productivity (€99.3 billion) and health-care costs (€37.0 billion) [6]. The prevalence of depression and its high costs explain why it is a major public health concern today [7,8].

The literature highlights that various risk factors for depressive episodes exist, including classically individual and biographical variables [9,10,11,12,13,14]. For instance, several studies show that they likely experience higher levels of socioeconomic disadvantage, resulting in more severe and chronic depression [15,16,17,18,19]. Additionally, it is widely reported in the literature that women are at greater risk for depression than men [15,16,20]; they also have more severe and longer episodes of depression than men.

More recently, biographical factors have been reported as affecting the risk of depression [13,21,22,23,24]. They include events that occur during childhood/adolescence (family disputes, serious parental disputes and sexual abuse) [23,24] or adulthood, such as limited mobility or a disability, an emotional or social breakdown (caused by major life events, such as bereavement, a break-up, job loss, home relocation, immigration or imprisonment), a suicide attempt, a negative body image and having experienced discrimination [10,11,12,14].

Beyond the above factors, contextual factors characterizing one’s residential neighborhood have also been reported to be related to adverse outcomes, including a depressive episode [25,26,27].

While these contextual factors can be measured from census data (for instance, to characterize people who live in low-income neighborhoods), other measures are more subjective, such as having a weak sense of belonging [28], feeling unsafe [29,30], having low mutual support, and having few relationships with neighbors [31]. A recent meta-analysis reported that adulthood depression is significantly higher among urban residents than in rural populations, except in China [32]. A study concluded that neighborhood characteristics, especially in the case of more disadvantaged neighborhoods, were associated with an increased risk of depression [33]. Weden et al. show a slight influence of neighborhood disadvantage on health (objective constructions) compared to the perceived neighborhood quality (subjective constructions) and stress the importance of taking both approaches into account [31]. It is important to note that women’s perceptions of a neighborhood are different from men’s [34].

In addition, several studies have reported the importance of considering the different areas frequented by an individual in the course of a day (beyond their residential neighborhood) to avoid estimation biases [19,35,36,37,38,39], with reference to the concept of “spatial polygamy” [39]. Presently, this is of particular interest, given people’s increased mobility, especially those living in urban areas. In Tucker et al.’s study, the risk of depression increased for individuals with low daily mobility: living in a more underprivileged neighborhood increased the risk of depression fourfold among the individuals with low mobility outside their residential neighborhood. However, this risk decreases among individuals with higher mobility, taking into account their demographics, their individual socioeconomic status and their functional limitation confounders [14].

In this context, one public health research question is this: does the combination of the various neighborhoods frequented by individuals influence the risk of mental health problems, and, more specifically, the risk of depression?

This present study aims to investigate this question in a population living in the Greater Paris area, which appears to be an ideal study location for many reasons. The capital of France, the Greater Paris area, covers 814 square kilometers and has 7 million inhabitants (approximately 10% of the French population). Paris is a world city, whose territory is marked by considerable socio-spatial segregation, which can be observed not only in residential areas, but also at all hours of the day [40,41]. The greatest segregation can be seen between two extremes of the social gradient: the wealthy elite and the most underprivileged social class [42,43], which is relegated to the most underprivileged neighborhoods in the northeastern suburbs of Paris. Previous work using the same data sources as this paper estimated that 12% of the adult population (18 years and older) in the Greater Paris area who showed signs of major depression were treated with antidepressants on the same day as the diagnosis [23]. This was significantly more common among those residing in poorer neighborhoods (17%).

The study is structured as follows:

- A description of the spatial distribution of depression in different neighborhoods in the Greater Paris area to identify places with a higher risk, and
- An investigation the role of individual and contextual perception of the neighborhood in the risk of depression and the socio-economic cumulative effect of different neighborhoods stratified for the overall population and stratified by gender.

## 2. Materials and Methods

### 2.1. Survey Design

This study is based on a cross-sectional analysis using data collected in the SIRS (a French acronym for “health, inequalities and social ruptures”) cohort study that involved a representative sample of French-speaking adults in the Paris metropolitan area. The overall objective of the cohort study was to investigate the relationships between individual, household and neighborhood social characteristics and health-related conditions. Data were collected during three waves, the first in 2005, the second in 2007 and the third in 2010. The analyses in the present study are based on data collected in 2010.

The SIRS survey employed a stratified, multistage cluster sampling procedure. The primary sampling units were census blocks, called “IRISs” (“IRIS” is a French acronym for “blocks for incorporating statistical information”). These are the smallest census spatial units in France (with about 2000 inhabitants each). In the SIRS survey, the Paris metropolitan area was divided into six strata according to the population’s socioeconomic profile [44], in order to over-represent the poorest neighborhoods. First, the census blocks were randomly selected within each stratum. In all, 50 census blocks were selected from the 2595 eligible census blocks in Paris and its suburbs. Second, within each selected census block, 60 households were randomly chosen from a complete list of households. Third, one adult was randomly selected from each household by the birthday method. Further details on the SIRS sampling methodology were previously published [45,46].

In our study, we used data collected on the 3006 people interviewed in the SIRS survey. A questionnaire containing numerous social and health-related questions was administered face-to-face during home visits.

### 2.2. Ethical Considerations

Legal authorization for the SIRS cohort study was obtained from two French authorities: the CCTIRS, (the French Advisory Committee on the Treatment of Information in Health Research) and the CNIL (the National Commission for Informatics and Liberties). The participants gave their verbal informed consent. Written consent was not necessary because this survey did not fall into the category of biomedical research (as defined by French law).

### 2.3. Outcome

Depression was assessed using the Mini-International Neuropsychiatric Interview (MINI) module pertaining to major depression, which is based on the Diagnostic and Statistical Manual of Mental Disorders-IV and International Classification of Diseases-10 criteria. The MINI has been used in many studies, and its validity has been well assessed [47]. The depression health outcome was recoded into a binary variable (yes/no).

### 2.4. Spatial Distribution of the Prevalence of Depression

To investigate the spatial distribution of the prevalence of depression by the mean yearly household income of 50 neighborhoods in the Greater Paris area, we created an IRIS spatial unit map. We classified the level of the prevalence of depression in neighborhoods in three categories (low (<11%), intermediary (11%–18%) and high (>18%)), based on Jenk’s method [48]. Mean yearly household income was estimated in euros per consumption unit and classified in three categories (low (<€6004/CU), intermediary (€6004/CU–€67,153/CU), high (>€67,153/CU)), based on Jenk’s method [48]. We used Arcgis 10 to do this mapping.

### 2.5. Study Variables

As described in the introduction, the factors that increase the risk of depression can be individual or contextual. The main hypothesis in this study was that depression was associated with certain individual characteristics, such as sociodemographic characteristics (low socioeconomic status) [9], negative perceptions (of oneself or of the neighborhood) [49,50] and difficult events [10]. We hypothesized that living in, working in and frequenting an deprived neighborhood (low income, low health-care density) and negative perceptions of the residential neighborhood were associated with depression [25,26,27]. 

#### 2.5.1. Individual and perception characteristics

Individual risk factors and confounders cover various dimensions: sociodemographic characteristics, social support, difficult life events (more details are provided in Appendix A).

Sociodemographic, social support and difficult events were detailed in Appendix D. Concerning the perception measures, it includes:

○ Activity space (large/not large) (more detail in Appendix B);
○ Feeling ashamed of his/her bodyweight: bodyweight perception (positive/negative);
○ Additionally, individual perception of his/her neighborhood of residence was collected through four binary variables: (i) the level of mutual aid between inhabitants (yes/no), (ii) feeling unsafe (yes/no), (iii) contact with neighbors (frequent/rare) and (iv) commercial density (sufficient/insufficient).

#### 2.5.2. Neighborhood Characteristics

In our study, we define three types of neighborhoods: (i) the residential neighborhood, the census block where the inhabitant was living during the study period; (ii) the workplace neighborhood, the workplace census block, plus the adjacent census blocks; and (iii) the frequented neighborhood, the census block frequented most regularly (after the residential and the workplace census blocks). Individuals were asked to give the neighborhood (address, postcode, and if not metro, train, bus, town, country) where they regularly went to meet relatives, for hobbies or other activities.

For each individual, in order to identify the three different types of neighborhoods, the reported postal address was converted as their corresponding IRIS in order to define the neighborhood (IRIS plus adjacent IRISs).

##### Income Level in the Three Types of Neighborhoods

We used the average income per consumption unit, estimated at the neighborhood level (defined as a measure of the residential IRIS plus the adjacent IRISs) using the data available from INSEE (French National Institute for Statistics and Economic Research) for 2010–2011. “Low”, “average” and “high” neighborhood income was defined according to the tertiles of the income distribution for all the IRISs that make up the Greater Paris area. If a participant did not have a job or did not declare a most frequented neighborhood, the income for these neighborhoods was considered missing and was classified in our results as “Not applicable”.

##### Aggregated Inhabitant Perceptions of the Residential Neighborhood

For inhabitant perceptions, we aggregated the answers from the 3006 interviewees from each residential neighborhood and constructed aggregated variables in order to capture the most widespread perception (negative or positive) for each census block.

Aggregated perceptions of a neighborhood were as follows: (i) commercial density (this included post offices, bakeries, banks, hairdressers, restaurants, beauty treatments, grocery stores, sports and hobby facilities, etc.) (insufficient/average/sufficient), (ii) the level of mutual aid between inhabitants (high/average/low), (iii) feeling unsafe (safe/somewhat safe/very unsafe) and (iv) contact with neighbors (frequent/occasional/rare).

In the following section, we will use the terms “residential neighborhood”, “workplace neighborhood” and “other frequented neighborhood” to refer to these three daily neighborhoods as previously defined.

##### Cumulative Exposure Score

One of the main original aspects of this study was to consider simultaneously the income levels in the three neighborhoods to better quantify the level of neighborhood socioeconomic deprivation. The underlying idea was to take into account the accumulation of the neighborhood socioeconomic deprivation with regard to the number of neighborhoods frequented (from one, only the residential neighborhood for an individual who worked and engaged in leisure-time activities in that neighborhood, for instance, to three, for an individual who moved between three different neighborhoods). More precisely, the accumulation of the neighborhoods’ characteristics, when these neighborhoods are frequented on a daily basis, may impact an individual’s health, including the risk of depression.

The cumulative exposure score combined the low-, middle- and high-income categories for the three reported neighborhoods. Next, this score was classified in three different groups (Table 1 shows the distribution of the score by category):

- Group 1 included individuals who only frequented poor neighborhoods (all three reported neighborhoods). In other words, for each neighborhood, the income was classified in the low category. This corresponds to maximum sociospatial relegation.
- Group 2 is the opposite of Group 1, as it included individuals who lived in, worked in and frequented (for various reasons) wealthy neighborhoods only.
- Group 3 includes a mixture of the different types of neighborhoods. Here, there is no clear pattern because the neighborhoods are a combination of the low-, middle- and high-income categories.

### 2.6. Statistical Analysis

The statistical analysis was structured in many successive steps. Significant confounders in studying the association between individual characteristics (sociodemographics, social isolation, mental health and life events) and depression were identified with univariate logistic regression.

First, we implemented a simple multilevel logistic model between depression and the individual perceptions and the cumulative exposure score, adjusting for all the sociodemographic and difficult event variables; it corresponds to model M1. Given negative life events could be associated to neighborhood characteristics [42,51], we have adjusted our models on difficult event variables.

**Figure 1:**
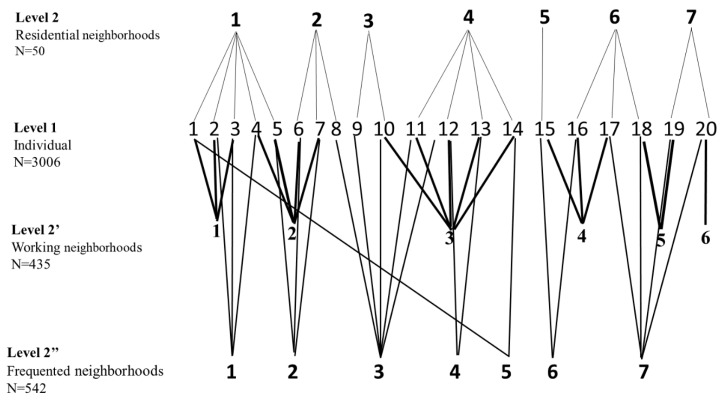
Cross-classified multilevel logistic models.

Second, a crossed-classified model was used to simultaneously account for the income level of the three types of neighborhoods (defining the model M2). This model enabled us to incorporate non-hierarchical nesting structures, where individuals were simultaneously nested within multiple non-hierarchical settings. The purpose of this step was to simultaneously examine the fixed and random effects, corresponding to the three types of neighborhoods. Thus, as shown Figure 1, in our cross-classified model, we included all the levels: Level 1 (individuals) and Level 2 (neighborhoods), the latter differentiated as follows: Level 2, the residential neighborhood; Level 2′, the workplace neighborhood; and Level 2′′, the other frequented neighborhood. We have detailed the theoretical framework of the cross-classified multilevel model in Appendix C.

As widely reported in the literature, women are at greater risk for depression, and have more negative perceptions of residential neighborhoods, than men [5,15,16,17,18,19,20,52]. We therefore performed a gender-stratified analysis (models M1W and M2W for women, and M1M and M2M for men).

All variation inflation factor (VIF) values to confirm collinearity between variables were below 2, not showing any specific issues regarding collinearity.

### 2.7. Statistical Implementation

All the statistical analyses were performed using R software and Bayesian estimation procedures. All the descriptive prevalences and proportions were weighted inversely to each participant’s inclusion probability, in accordance with the sampling design, with the “survey” package. We implemented a logistic regression to investigate our binary outcome: depression (yes/no). Model fit was assessed using the deviance information criterion (DIC), a Bayesian measure of model fit (analogous to Akaike Information Criterion in frequentist statistics). This test statistic yielded by the procedure assesses how well the model fits the data, with a penalty on model complexity, and is referred to as a “badness of fit” indicator. Therefore, higher DIC values indicate a poorer-fitting model.

## 3. Results

### 3.1. Description of Population

The gender ratio (M/F) was 0.65, and the average age was about 45 years. Most of the participants had a postsecondary level of education (31.2%), and 12.6% were foreigners. Of the total participants, 41.3% were single, and 53.6% were employed. Their average monthly income was €2014 per consumption unit. On average, the proportion of depression was 14.3% in the total population and 10.5% and 16.7% for men and women, respectively.

### 3.2. Spatial Distribution of Depression

**Figure 2:**
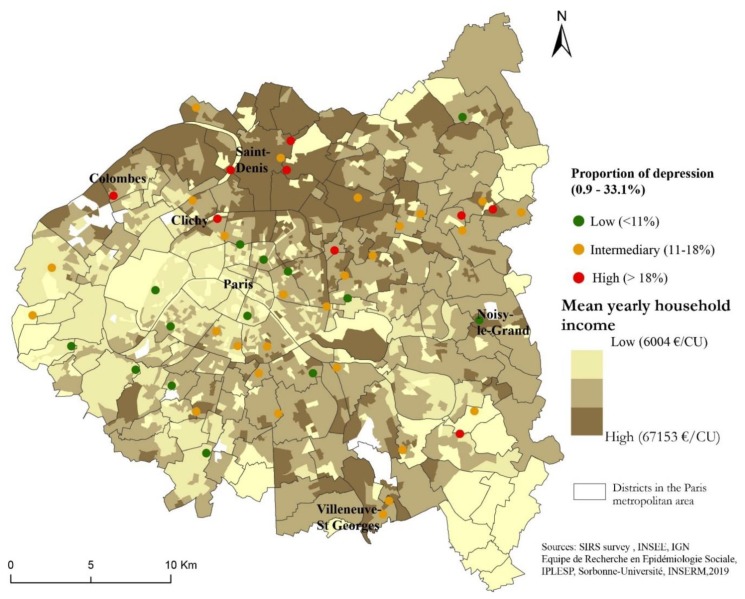
Spatial distribution of the proportion of depression by category in the 50 residential neighborhoods in the SIRS survey (a French acronym for “health, inequalities and social ruptures”), Greater Paris area, 2010.

The proportion of individuals with depression varied between a minimum of 0.9% and a maximum of 33.1% for the 50 residential neighborhoods in the SIRS survey (Figure 2). Depression was reported more frequently in the most deprived neighborhoods (those with a yearly household income <€6004 by consumption unit on average), which are located in the northern part of the study area, than in the < most privileged neighborhoods (those with a yearly household income >€67,153 by consumption unit on average), which are located in the center of Paris and in the western part of the study area.

Cumulative exposure score: we observed that most of the participants (73.6%) frequented neighborhoods of different types, while 17.9% had frequented only poor ones, and 8.4% had frequented only wealthy ones (Table 1). The cumulative exposure score was significantly associated with depression (OR = 2.87; % 95% CI = [1.77–4.64]) (Table 2).

### 3.3. Individual Factors Associated with Depression (Univariate Analysis)

As shown by Appendix D Table A1, the prevalence of depression was higher in women (OR = 1.71; 95% CI = [1.24–2.34]), and individuals with a low monthly household income (OR = 1.81; 95% CI = [1.16–2.83]), compared to those with a higher income, those who were unemployed (OR = 3.35; 95% CI = [1.68–3.29]) and compared to those who were active, those with perceived social isolation (OR = 5.58; 95% CI = [4.38–7.12]), handicapped or disabled individuals (OR = 4.98; 95% CI = [3.76–6.59]), individuals who had a relative or close friend with a serious disease (OR = 1.43; 95% CI = [1.11–1.84]), and those who had attempted suicide before the age of 18 years (OR = 5.23; 95% CI = [2.82–9.72]).

### 3.4. Contextual Factors Associated with Depression

According to Table 2, contextual factors associated with depression were: low mutual aid between inhabitants (OR = 3.64; 95% CI = [1.92–6.92]), feeling very unsafe (OR = 1.85; 95% CI = [1.32–2.60]), not having regular contact with neighbors (OR = 1.51; 95% CI = [1.08–2.60]), feeling very unsafe (OR = 1.62; 95% CI = [1.09–2.43]), residing in low and/or average neighborhoods (OR = 2.36; 95% CI = [1.57–3.54]) and (OR = 2.16; 95% CI = [1.43–3.25]).

### 3.5. Individual Perceptions Measures

Table 3 highlights that the following aggregated variables concerning neighborhood perception were associated with a higher prevalence of depression: bodyweight perception negative (OR = 1.38; 95% CI = [1.00–1.91]), feeling very unsafe (OR = 1.62; 95% CI = [1.09–2.43]), perceiving an insufficient commercial density within their neighborhood (OR = 1.38; 95% CI = [1.02–1.86]), and individuals who frequented neighborhoods of different types (OR = 2.0; 95% CI = [1.32–3.29]) and who frequented only poor neighborhoods (OR = 1.72; 95% CI = [1.16–2.55]).

### 3.6. Contextual Factors Associated with Depression

According to Table 3, the prevalence of depression was higher in individuals residing in a neighborhood with a low and average household monthly income (respectively, OR = 1.91, 95% CI = [1.07–3.42]; OR = 2.02, 95% CI = [1.14–3.57]), compared to those living in a neighborhood with a high household monthly income. However, the prevalence of depression was not significantly different in individuals in a workplace or frequented neighborhood with a low or average household monthly income, compared to the neighborhoods with a higher household monthly income.

Models 1 (M1) and 2 (M2) were adjusted for individual characteristics (gender, monthly household income, employments status, relationship status, perceived social isolation, handicapped or disabled, serious illness or friend/family member, serious familial disputes before 18, sexual abuse during childhood, attempted suicide before 18).

### 3.7. Comparison between Women and Men

We will focus now on the differences that can exist between women and men.

Among women, Table 4 reveals that the individual perceptions measures associated with depression were: bodyweight perception negative (OR = 1.44; 95% CI = [1.03–2.01]), perceived an insufficient commercial density within their neighborhood (OR = 1.41; 95% CI = [1.02–1.96]) and the women who frequented neighborhoods of different types (OR = 1.51; 95% CI = [1.04–2.17]). The contextual characteristics associated with depression among women were the ones who resided in a neighborhood with a low and average household monthly income (respectively, OR = 2.50, 95% CI = [1.10–5.67]; OR = 2.18, 95% CI = [1.05–4.50]).

Among men, Table 4 reveals that the individual perceptions measures associated with depression were: felt unsafe (OR = 2.23; 95% CI = [1.14–4.38]), did not have regular contact with their neighbors (OR = 2.48; 95% CI = [0.95–6.51]), and the men who frequented neighborhoods with cumulative poverty (OR = 3.69; 95% CI = [1.03–13.25]). The only contextual characteristics associated with depression among men is to feel unsafe (OR = 4.57; 95% CI = [2.04–10.27]).

Models 1 and 2 of women (M1W and M2W, respectively) and men (M1M and M2M, respectively) were adjusted for individual characteristics (monthly household income, employments status, relationship status, perceived social isolation, handicapped or disabled, serious illness or friend/family member, serious familial disputes before 18, sexual abuse during childhood, attempted suicide before 18).

## 4. Discussion

### 4.1. Main Findings

In this study, the prevalence of depression was higher among people living in poor neighborhoods. Furthermore, after adjusting for individual characteristics and difficult life events, this study indicated that depression was associated with a negative perception of one’s bodyweight, feeling unsafe and a perception of one’s neighborhood as being deprived, in terms of income and available services. There is also a higher risk of depression among people who frequented only poor and/or mixed neighborhoods.

### 4.2. Comparison with Previous Studies

Our study confirm previous classical finding regarding the individuals’ risk factors of depression. Whereas numerous previous studies have shown that certain neighborhood characteristics, such as income and a built environment, may be associated with a higher ‘ecological’ risk of depression, this study is the first one in France to consider the contextual characteristics of both residential and nonresidential neighborhoods in multilevel models, that take into account individual characteristics and/or perceptions of their residential neighborhood.

Comparing our results with those found in the literature is difficult because of differences in the contextual characteristics examined in each study. Most studies agree that neighborhood income level has a significant influence on depression [26,27,53]. Neighborhood income could be consider as a proxy for other neighborhood characteristics, such as social cohesion, safety, the services offered or a built environment (not to mention biases in the socio-economic neighborhood when used without adjustment for the inhabitants’ individual characteristics in a merely ecological analysis). A study showed that people residing in the most underprivileged neighborhoods had a higher risk of depression than those living in privileged neighborhoods [52]. Some studies suggested that the esthetic quality of a neighborhood (such as introducing more appealing elements, such as green spaces, in order to create a pleasant environment) could be associated with people’s health [54]. However, Burt et al. showed that access to green spaces was only beneficial to men’s mental health and that it varied with age [54]. Furthermore, Choi et al. showed that feeling unsafe within a residential neighborhood significantly increased the risk of depression [29]. In addition, they found that people who lived in neighborhoods with strong social cohesion are more likely to have a stronger sense of belonging, which, in turn, can have an influence on their health. Finally, studies have also shown that certain difficult life and contextual factors are associated with a higher risk of depression [22,27,53].

The neighborhood characteristics for women with depression include a low household income, a negative self-image, feeling different from their neighbors, a low density of services in the area, and residing in a low- or average-income neighborhood. These results could be partially explained by the fact that women tend to have a more negative and more selective representation than men. This difference between men and women increases when focusing the analysis among women with children because:

- mothers are typically the main household manager in a family’s daily life [22];
- women depend more on emotional support and personal relationships in which emotional intimacy, trust and solidarity are exchanged than men [19];
- a disadvantaged socio-economic situation may therefore be the main explanation for the higher level of depression among women [17].

Some models showed that the gender gap in depression could also be due to higher exposure of difficult events [19].

The last point concerns the socioeconomic diversity score. To our knowledge, no study constructing this type of score has been constructed and used. Of course, for future surveys, the score could be improved to better identify individuals’ frequented destinations, in addition to the three neighborhoods of interest in this study, and/or look at others socioeconomic characteristics other than the neighborhood’s average household income. In addition, we recognize that the score estimated for people included in the group 3 could characterize various socioeconomic situations. Indeed, whereas the score estimated for people included in group 1 and group 2 could reflect their individual socioeconomic position, for those in group 3, it would provide an incorrect assessment of their socioeconomic position. For instance, people may have a high individual socioeconomic position (as a general practitioners of a lawyer) but working in a poor neighborhood, and inversely. To improve the interpretation of people classified in group 3, it would be appropriate to combine the socioeconomic diversity score with information on occupational status. However, this information was not available in the SIRS cohort.

### 4.3. Limitations and Strengths

First, we note a limitation concerning the existence of inter-individual variability in defining a “neighborhood” and its boundaries [23]. Using the residential neighborhood as an example, the boundaries and area of a perceived neighborhood vary from one individual to another (it was observed that they were perceived to be smaller in the inner city if Paris than in the suburbs) [23,24]. In addition, the responses may have varied according to the manner in which the questions were worded. A person could delimit his/her neighborhood of residence as a small building block around his/her apartment building when defining his/her built environment (e.g., visible from its windows), but then widen the space when assessing the density of shops or services accessible by foot.

Second, there was a possibility of same-source bias, because the outcome affects the perception the neighborhood attribute. However, previous studies indicated that the aggregation of the responses of the same neighborhood, as we did, permit to reduce the same-source bias because the measurement error in individuals’ responses was averaged [53,55] 

Thirdly, there was a possibility of self-selection bias. The self-selection bias concerns the predisposition (i) of people to settle in different neighborhoods from their wishes (the most precarious) (ii) and certain people to be able to choose their neighborhood (the most affluent) [55]. James et al. claim that sometimes these constraints can lead some individuals with a high BMI to move to neighborhoods that have lower density and accessibility.

The measures of association with depression could be affected if individuals with depression are “more likely to live” in underprivileged neighborhoods and, conversely, if individuals in good mental health are “more likely to live” in more advantaged neighborhoods. Julie Vallée et al. showed that people with depression are more likely to report that their residential neighborhood has problems or a low level of social cohesion [23,24]. These biases are not incurred when objective indicators (from census data or household income tax data), or combined aggregate-level subjective neighborhood data, are used.

This study has some strengths. First, we have a representative sample of the Paris metropolitan area which takes into account these specificities [56]. Second, in our study, defining a “neighborhood” (residential, workplace or frequented) was left to the respondent’s judgment. Despite the fact that, for the purposes of the analysis, all the neighborhoods were redefined using the address provided by the individual and then grouped by IRIS and adjacent IRISs, on average, the neighborhoods were 2.55-km^2^ polygons, with a population of 16,305 inhabitants. This systematization simplifies the diversity of the situations observed and reported in the literature [57]. Third, the SIRS survey contains various variables, which allowed it to take into account the complexity of the mechanisms of depression, and the relationship between individual and contextual factors.

## 5. Conclusions

Our study confirmed the existence of a significant association between the socioeconomic status of a residential neighborhood and depression. It also highlighted a gender-modifying effect when measuring the association between residential factors and depression. For women, self-help and a neighborhood’s average monthly household income were significantly associated with depression, while for men, only feeling unsafe was significant. This study also showed the importance of a cumulative approach to socioeconomic diversity in the multiple contextual characterization of individuals when considering their multiple frequented spaces. This score can be considered as an alternative approach to analyze the effects of contextual characteristics in the investigation of socio-territorial inequalities in health. However, the contextual effect of the three combined neighborhoods could be improved if the relative time spent within each neighborhood were measured, which would permit a more complete study on the impact of an individual’s contextual exposure and his/her risk of depression [58,59]. For future research in this area, one interesting challenge to consider would be to shift towards more dynamic forecasts, by using short-term time scales. A solution to this problem is found in the latest methodological advances, which make it possible to examine a variable place as a function of time and duration, in order to better characterize exposure to the different environments frequented and traversed by individuals [60]. For example, neighborhood mobility factors may play a role in the period in which participants are exposed to impoverished contexts, which may, in turn, influence their susceptibility to react negatively to daily stressors. Others studies suggest finding an alternative to help identify a critical or sensitive time period in which a person may be exposed to daily stressors in a neighborhood [27,61].

## Figures and Tables

**Table 1:** Distribution of cumulative exposure scores.

Category	*n*	%
Group 1: Poor neighborhoods only	254	12.1
Group 2: Wealthy neighborhoods only	539	9.1
Group 3: Neighborhoods of different types	2213	78.8

**Table 2:** Univariate analysis of the associations between contextual neighborhood characteristics and depression, SIRS, 2010.

Contextual Characteristics	*N*	Percentage	Depression	OR 95% [CI]	*p*-Value
Mutual aid between inhabitants in RN					**0.001**
Low	119	3.7	25.8	3.64 [1.92–6.92]	
Average	2647	88.5	13.7	1.67 [0.93–3.00]	
High	240	7.8	8.7	Ref.	
Feeling unsafe in RN					**0.002**
Safe	1138	45.1	11.3	Ref.	
Somewhat safe	1203	40.9	14.8	1.37 [1.01–1.85]	
Very unsafe	665	14.0	19.0	1.85 [1.32–2.60]	
Contact with neighbors in RN					0.034
Frequent	2105	75.5	12.8	Ref.	
Occasional	602	15.7	16.1	1.30 [0.94–1.81]	
Rare	299	8.8	18.1	1.51 [1.08–2.12]	
Commercial density in RN					0.500
Insufficient	1500	60.5	13.0	0.85 [0.52–1.40]	
Average	1084	30.7	15.0	1.00 [0.61–1.66]	
Sufficient	422	8.8	14.9	Ref.	
Income level in RN					**0.001**
High	479	32.8	7.5	Ref.	
Average	1198	47.6	14.8	2.16 [1.43–3.25]	
Low	1329	19.7	16.0	2.36 [1.57–3.54]	
Income level in WN					**0.001**
High	325	14.3	9.1	Ref.	
Average	737	30.9	11.4	1.29 [0.73–2.29]	
Low	600	17.4	10.4	1.16 [0.64–2.09]	
Not applicable	1344	34.3	19.1	2.36 [1.40–3.96]	
Income level in FN					0.956
High	680	25.3	13.7	Ref.	
Average	754	27.1	13.7	1.00 [0.73–1.37]	
Low	376	10.7	14.9	1.10 [0.71–1.72]	
Not applicable	1196	36.9	13.5	0.99 [0.74–1.32]	

RN: residential neighborhood; WN: workplace neighborhood; FN: frequented neighborhood. OR: odds ratio, CI: confidence interval, Ref.: reference group. In bold, these are statistically significant results at the threshold of a *p*-value of 0.05 or less.

**Table 3:** Multivariate analysis of the associations between individual perceptions measures, cumulative exposure score, contextual neighborhood characteristics and depression, SIRS, 2010.

	M1	M2
	OR 95% [CI]	OR 95% [CI]
Individual perception measures		
Bodyweight perception	**0.051**	**0.051**
Positive	Ref.	Ref.
Negative	1.38 [1.00–1.91]	1.37 [1.00–1.90]
Mutual aid between inhabitants	0.430	0.574
Yes	Ref.	Ref.
No	0.89 [0.68–1.18]	0.86 [0.65–1.15]
Feeling unsafe	**0.018**	**0.009**
No	Ref.	Ref.
Yes	1.62 [1.09–2.43]	1.62 [1.08–2.44]
Contact with neighbors	0.314	0.129
Frequent	Ref.	Ref.
Rare	1.32 [0.77–2.29]	1.44 [0.83–2.49]
Commercial density	**0.035**	**0.033**
Sufficient	Ref.	Ref.
Insufficient	1.38 [1.02–1.86]	1.45 [1.09–1.93]
Cumulative exposure score		
Wealthy neighborhoods only	**0.005**	
All types of neighborhoods	1.72 [1.16–2.55]	
Poor neighborhoods only	2.08 [1.32–3.29]	
Contextual measures		
Mutual aid between inhabitants		**0.042**
Low		1.71 [0.83–3.69]
Average		0.92 [0.51–1.78]
High		Ref.
Feeling unsafe		0.284
Safe		Ref.
Somewhat safe		1.04 [0.75–1.43]
Very unsafe		1.50 [0.89–2.52]
Contact with neighbors		0.145
Frequent		Ref.
Occasional		0.83 [0.51–1.33]
Rare		1.25 [0.94–1.66]
Commercial density		0.372
Insufficient		Ref.
Average		1.16 [0.76–1.77]
Sufficient		1.46 [0.94–2.26]
Residential neighborhood		**0.043**
High		Ref.
Average		2.02 [1.14–3.57]
Low		1.91 [1.07–3.42]
Workplace neighborhood		0.056
High		Ref.
Average		1.05 [0.56–1.97]
Low		0.64 [0.35–1.18]
Not applicable		1.49 [0.50–4.38]
Frequented neighborhood		0.301
High		Ref.
Average		0.86 [0.61–1.22]
Low		0.80 [0.52–1.21]
Not applicable		0.80 [0.56–1.14]

OR: Odds Ratio, CI: Confidence Interval, Ref.: reference group. In bold, these are statistically significant results at the threshold of a *p*-value of 0.05 or less.

**Table 4:** Multivariate analysis of the associations between contextual neighborhood characteristics and depression in women and men, SIRS, 2010.

	Women		Men	
	M1W	M2W	M1M	M2M
	OR 95% [CI]	OR 95% [CI]	OR 95% [CI]	OR 95% [CI]
Individual perception measures				
Bodyweight perception	**0.033**	**0.016**	0.404	0.598
Positive	Ref.	Ref.	Ref.	Ref.
Negative	1.44 [1.03–2.01]	1.54 [1.16–2.04]	1.24 [0.75–2.04]	1.39 [0.84–2.30]
Mutual aid between inhabitants	0.816	0.726	0.272	0.864
Yes	Ref.	Ref.	Ref.	Ref.
No	0.96 [0.70–1.32]	0.99 [0.74–1.32]	0.74 [0.43–1.27]	0.87 [0.51–1.49]
Feeling unsafe	0.122	0.064	**0.020**	**0.022**
No	Ref.	Ref.	Ref.	Ref.
Yes	1.44 [0.91–2.30]	1.46 [0.95–2.24]	2.23 [1.14–4.38]	2.25 [1.29–3.95]
Contact with neighbors	0.982	0.373	0.064	0.195
Frequent	Ref.	Ref.	Ref.	Ref.
Occasional	1.01 [0.53–1.93]	1.16 [0.63–2.11]	2.48 [0.95–6.51]	1.65 [0.70–3.90]
Commercial density	**0.039**	0.072	0.431	0.277
Sufficient	Ref.	Ref.	Ref.	Ref.
Insufficient	1.41 [1.02–1.96]	1.36 [0.99–1.86]	1.26 [0.71–2.26]	1.34 [0.80–2.24]
Cumulative exposure score	**0.087**		**0.057**	
Wealthy neighborhoods only	Ref.		Ref.	
All types of neighborhoods	1.51 [1.04–2.17]		1.95 [0.57–6.65]	
Poor neighborhoods only	1.34 [0.73–2.45]		3.69 [1.03–13.25]	
Individual perception measures				
Mutual aid between inhabitants		**0.009**		0.836
Low		1.40 [0.53–3.64]		1.71 [0.50–5.88]
Average		0.64 [0.31–1.32]		1.52 [0.54–4.29]
High		Ref.		Ref.
Feeling unsafe		0.884		**0.009**
Safe		Ref.		Ref.
Somewhat safe		0.89 [0.55–1.44]		1.35 [0.79–2.31]
Very unsafe		0.83 [0.35–1.98]		4.57 [2.04–10.27]
Contact with neighbors		0.896		0.068
Frequent		Ref.		Ref.
Occasional		1.18 [0.67–2.08]		0.43 [0.23–0.81]
Rare		1.01 [0.55–1.85]		1.43 [0.77–2.65]
Commercial density		0.839		0.211
Insufficient		0.94 [0.52–1.72]		1.06 [0.54–2.09]
Average		0.92 [0.49–1.74]		1.10 [0.46–2.65]
Sufficient		Ref.		Ref.
Residential neighborhood		**0.029**		0.731
High		Ref.		Ref.
Average		2.18 [1.05–4.50]		1.57 [0.56–4.41]
Low		2.50 [1.10–5.67]		1.25 [0.43–3.64]
Workplace neighborhood		0.883		**0.001**
High		Ref.		Ref.
Average		0.85 [0.45–1.61]		1.32 [0.41–4.28]
Low		0.84 [0.47–1.51]		0.37 [0.11–1.26]
Not applicable		0.78 [0.25–2.40]		2.06 [0.77–5.48]
Frequented neighborhood		0.346		**0.015**
High		Ref.		Ref.
Average		1.39 [0.89–2.17]		0.52 [0.23–1.14]
Low		0.98 [0.54–1.79]		0.69 [0.35–1.37]
Not applicable		0.96 [0.58–1.59]		0.72 [0.36–1.43]

OR: Odds Ratio, CI: Confidence Interval, Ref.: reference group. In bold, these are statistically significant results at the threshold of a p-value of 0.05 or less.

## Appendix A. Variables Definition

### Appendix A.1.1. Construction of the Variable “Perceived Social Isolation”

The question was “Generally speaking, did you feel very alone, rather alone, rather supported or very supported?” Participants who answered “very or rather alone” were classified in “perceived social isolation” and participants who answered “rather or very supported” were classified in “not perceived social isolation”.

### Appendix A.1.2. Construction of the Variable “Ashamed of One’s Bodyweight”

Step 1: After showing the participants images of different silhouettes, we asked them the following question to see which one corresponds to them the best.

**Presently, Would You Prefer****To**	**Response**
Maintain your current weight	1
Lose weight	2
Gain weight	3
It doesn’t matter to me	4

Step 2: We placed those who wanted to gain or lose weight in the category “negative perception of their build” and the others in the category “positive perception of their build”.

### Appendix A.1.3. Formulation of the Questions about the Neighborhood

**With Regard to Your Neighborhood, Please Reply to the Following Statement and Questions**	**Possible Answers**
People who live there readily help each other	Yes/no
Do you personally feel unsafe in your neighborhood?	Yes/no
Apart from simple greetings, how often do you have face-to-face contact with neighbors, shopkeepers or other people in your neighborhood?	More often than not/Rarely
Do you think there are enough businesses and miscellaneous stores in your neighborhood (commercial density)?	Yes/no

## Appendix B. Definition of the Activity Space Score

In this paper, activity space was measured from the respondents’ statements about the location of their domestic and social activities. In the SIRS survey, people were asked where they usually (1) went food shopping; (2) used services (e.g., bank and post office); (3) went for a walk; (4) met friends; and (5) went to a restaurant. For each of these five activities, there were three answer options: (1) mainly within your residential neighborhood; (2) mainly outside your residential neighborhood; and (3) both within and outside your residential neighborhood.

An activity space measure was subsequently created: activities said to be done mainly within the neighborhood were assigned a value of 1, while those done both within and outside the neighborhood or mainly outside the neighborhood were assigned a value of 0.5 and 0, respectively. By adding these values together and dividing the sum by the total number of reported activities, we obtained an individual score, measuring the concentration of daily activities in the perceived neighborhood. The respondents were then ranked on the basis of this score (variable called “final score”). It ranged from 0 (for individuals who reported doing all the activities of interest mainly outside their neighborhood of residence) to 1 (for people who reported doing all the activities of interest, mainly within their neighborhood of residence), and can be considered a proxy of personal exposure to the residential neighborhood [23].

## Appendix C. Theoretical Framework of the Cross-Classified Multilevel Model

Theoretically, in a cross-classified multilevel model, an individual (i) simultaneously belongs to three non-nested contexts, here the residential neighborhood (j), the workplace neighborhood (h) and another frequented neighborhood (k). Thus, since our outcome (Y) is a binary variable, the probability of depression for an individual i living in a residential neighborhood j and travelling to a workplace neighborhood h and another frequented neighborhood *k* is modeled in null or intercept-only (i.e., a model without covariates) regression as follows:

Logit (II_i(jhk)_) = β_0_ + μ_0j_ + μ_0h_ + μ_0k_ + ε_0(jhk)_

where:

- the fixed effect parameter, β_0_, refers to the overall probability mean of the outcome Y across all residential, workplace and frequented neighborhoods,
- μ_0j_ refers to the random effect for residential neighborhoods, μ_0h_ for workplace neighborhoods and μ_0k_ for other frequented neighborhoods, and ε_0(jhk)_ to the random effect for the individual with the combination of j residential neighborhood, h workplace neighborhood and k other frequented neighborhood.

Therefore, in this model, we compared the relative variance contribution of residential neighborhood, workplace neighborhood, frequented neighborhood, by comparing variance contributions (i.e., random effects) across models. The null model describe in the equation above can be extended to include covariates (i.e., fixed effects) at each level of analysis; it corresponds to the previously defined model M2.

## Appendix D. Individual Social Support and Difficult Events

**Table A1:** Univariate analysis of the association between individual characteristics, individual perception of neighborhood, and the cumulative exposure score and depression, SIRS, 2010.

	N	Percentage	Depression	OR 95% [CI]	*p*-Value
**Gender**					**0.001**
Man	1187	46.9	10.5	Ref.	
Woman	1819	53.1	16.7	1.71 [1.24–2.34]	
**Age**					0.398
18–29 years	208	14.3	12.5	Ref.	
30–44 years	796	30.5	12.4	0.99 [0.62–1.58]	
45–59 years	857	26.6	15.2	1.26 [0.82–1.94]	
60 and over	1145	28.6	14.5	1.19 [0.77–1.82]	
**Nationality**					0.370
French	2002	66.6	13.2	Ref.	
Mixed	610	20.8	16.1	1.26 [0.91–1.74]	
Foreigner	394	12.6	12.9	0.97 [0.66–1.42]	
**Monthly household income (€/CU)**					**0.001**
<1116	855	25.0	19.9	2.35 [1.68–3.29]	
1116–1733	764	24.8	14.1	1.56 [1.17–2.09]	
1734–2605	714	25.3	11.6	1.24 [0.86–1.80]	
≥2606	673	25.0	9.5	Ref.	
**Employment status**					**0.001**
Active	1596	56.7	10.9	Ref.	
Student	111	7.7	9.4	0.85 [0.40–1.78]	
Unemployed	212	7.6	29.1	3.35 [2.06–5.45]	
Retired	796	19.8	15.4	1.48 [1.14–1.93]	
Inactive	265	7.3	19.2	1.94 [1.23–3.08]	
**Social support**					
**Relationship status**					**0.001**
Living with partner	1766	64.3	10.9	Ref.	
Not living with partner	1240	35.7	18.9	1.91 [1.48–2.47]	
**Perceived social isolation**					**0.001**
Isolated	2469	86.5	10.0	Ref.	
Not isolated	525	13.2	38.4	5.58 [4.38–7.12]	
**Difficult life events**					
**Handicapped or disabled**					**0.001**
No	2648	91.0	11.3	Ref.	
Yes	358	9.0	38.8	4.98 [3.76–6.59]	
**Friend/family member with a serious disease**					**0.001**
No	1524	49.7	11.9	Ref.	
Yes	1413	47.3	16.3	1.45 [1.16–1.8]	
**Serious familial disputes before age 18**					**0.001**
No	2405	81.4	12.6	Ref.	
Yes	601	18.6	19.0	1.63 [1.29–2.06]	
**Attempted suicide before age 18**					**0.001**
No	2881	96.5	12.4	Ref.	
Yes	125	3.5	50.2	5.23 [2.82–9.72]	
**Sexual abuse during childhood**					**0.002**
No	2884	96.9	27.8	2.51 [1.38–4.55]	
Yes	122	3.1	13.3	Ref.	
**Had been in prison**					0.135
No	2935	97.9	12.4	Ref.	
Yes	71	2.1	50.2	2.09 [0.79–5.49]	
**Individual perception measures**					
**Activity space**					0.996
Large	2439	81.2	13.8	1.00 [0.71–1.42]	
Not large	567	18.8	13.8	Ref.	
**Bodyweight perception**					**0.001**
Positive	1510	49.8	10.9	Ref.	
Negative	1496	50.2	16.7	1.64 [1.27–2.12]	
**Mutual aid between inhabitants**					0.147
Yes	1523	50.9	12.9	Ref.	
No	1483	49.1	14.7	1.71 [0.95–1.44]	
**Feeling unsafe**					**0.001**
No	2361	82.3	11.9	Ref.	
Yes	645	17.7	22.6	2.16 [1.52–3.08]	
**Contact with neighbors**					**0.001**
Frequent	2606	86.6	13.4	Ref.	
Rare	400	13.4	13.8	1.62 [1.31–2.00]	
**Commercial density**					**0.002**
Insufficient	745	19.4	18.2	1.52 [1.17–1.98]	
Sufficient	2261	80.6	12.7	Ref.	
**Cumulative exposure score**					**0.001**
Wealthy neighborhoods only	539	12.1	7.7	Ref.	
All types of neighborhoods	2213	78.8	13.6	1.88 [1.19–2.98]	
Poor neighborhoods only	254	9.1	19.4	2.87 [1.77–4.64]	

OR: Odds Ratio, CI: Confidence Interval, Ref.: reference group. In bold, these are statistically significant results at the threshold of a *p*-value of less than 0.05.
